# A hardware model of the auditory periphery to transduce acoustic signals into neural activity

**DOI:** 10.3389/fneng.2013.00012

**Published:** 2013-11-26

**Authors:** Takashi Tateno, Jun Nishikawa, Nobuyoshi Tsuchioka, Hirofumi Shintaku, Satoyuki Kawano

**Affiliations:** ^1^Special Research Promotion Group, Graduate School of Frontier Biosciences, Osaka UniversityOsaka, Japan; ^2^Biomedical Systems Engineering, Bioengineering and Bioinformatics, Graduate School of Information Science and Technology, Hokkaido UniversitySapporo, Japan; ^3^Graduate School of Engineering Science, Osaka UniversityOsaka, Japan; ^4^Department of Micro Engineering, Graduate School of Engineering, Kyoto UniversityKyoto, Japan

**Keywords:** acoustic sensor, computer model, digital signal processor, piezoelectric film, electrically evoked auditory brainstem response

## Abstract

To improve the performance of cochlear implants, we have integrated a microdevice into a model of the auditory periphery with the goal of creating a microprocessor. We constructed an artificial peripheral auditory system using a hybrid model in which polyvinylidene difluoride was used as a piezoelectric sensor to convert mechanical stimuli into electric signals. To produce frequency selectivity, the slit on a stainless steel base plate was designed such that the local resonance frequency of the membrane over the slit reflected the transfer function. In the acoustic sensor, electric signals were generated based on the piezoelectric effect from local stress in the membrane. The electrodes on the resonating plate produced relatively large electric output signals. The signals were fed into a computer model that mimicked some functions of inner hair cells, inner hair cell–auditory nerve synapses, and auditory nerve fibers. In general, the responses of the model to pure-tone burst and complex stimuli accurately represented the discharge rates of high-spontaneous-rate auditory nerve fibers across a range of frequencies greater than 1 kHz and middle to high sound pressure levels. Thus, the model provides a tool to understand information processing in the peripheral auditory system and a basic design for connecting artificial acoustic sensors to the peripheral auditory nervous system. Finally, we discuss the need for stimulus control with an appropriate model of the auditory periphery based on auditory brainstem responses that were electrically evoked by different temporal pulse patterns with the same pulse number.

## Introduction

To treat some types of hearing loss, cochlear implants (CIs) have been used to directly generate electric currents in auditory nerve (AN) fibers, bypassing damaged hair cells and compensating for dysfunction in the inner ear (for review, Eisen, 2000; Møller, 2006). Although these implants are one of the great success stories of modern biomedical engineering, they are not without limitations—e.g., the need for extracorporeal devices, the paucity of electrodes compared with the number of AN fibers, and significant power requirements. These problems contribute to a wide variation in the efficacy of these devices among treated individuals, and have motivated additional efforts to improve the performance of the hardware (Niparko, 2009).

All current CI systems use some form of an auditory model. For instance, filter banks are used to mimic the filtering that occurs in the *in vivo* auditory periphery. These models, however, are simple relative to the complexity of normal auditory processing, although the auditory periphery is not a simple biological way to generate a spectral analysis of an acoustic signal input.

Processing in the peripheral auditory system begins when a pressure wave enters the ear, and ends with a neural signal in the AN. Because each successive transformation of the signal in the auditory periphery contributes to the quality of hearing, all of these processes should be included in any computational model of auditory peripheral processing. To accomplish this goal, computer models have been developed using a series of auditory processing stages leading to the transfer of auditory information to the AN. The more recent models are markedly complex (Meddis and Lopez-Poveda, 2010) and some have been adopted for practical applications (van Schaik and Meddis, 1999). For example, Carney and colleagues proposed a composite phenomenological model of AN responses that reproduces a large number of non-linear AN response characteristics (Heinz et al., 2001; Zhang et al., 2001; Tan and Carney, 2003). Additionally, Meddis and coworkers proposed a non-linear filter model of the basilar membrane (BM), which can predict AN representations of stimuli with complex spectra, speech, harmonic complexes, and amplitude-modulated stimuli (Lopez-Poveda and Meddis, 2001; Meddis et al., 2001; Ferry and Meddis, 2007); of note, this model has been used to build a speech processor for CIs (Wilson et al., 2005).

In this study, to obtain AN responses, we developed a phenomenological model of the auditory periphery, which requires relatively simple hardware. To implement the model, a biomimetic mechanical device was used as an acoustic sensor that mimics auditory peripheral signal processing and structures, because developing a small, low-power, analog, and multiresonator system that functionally replaces the cochlea is critical step toward an implantable completely bionic ear. In this report, we have focused on micromechanical devices as bionic acoustic sensors (BASs) and briefly describe how to use BAS devices to mimic frequency selectivity in the cochlea. In the processing stages, however, our model did not include active feedback mechanisms that enhance passive BM filtering and cause non-linearities in the responses to small auditory signals. Therefore, a restricted range of acoustic parameters [i.e., moderate to high sound pressure levels (SPLs) and frequencies] was examined using the proposed model. Next, to integrate inner hair cells (IHCs), IHC-AN synapses, and AN fibers, we developed a simple computer model with the BAS and simulated the responses of AN fibers with high spontaneous firing rates. Thus, we examined information encoding and processing in the peripheral auditory system by developing a hybrid model containing a BAS device and a digital signal processor (DSP) for real-time signal processing. In addition to better modeling, improved replication of the precise and interactive processing that occurs in the auditory periphery depends on the degree of stimulus control. Moreover, to develop better CIs, the final transformation stage from electrical stimulation to evoked responses in AN fibers must be well understood. Thus, we used simple temporal patterns of electrical pulses to stimulate the cochleae of guinea pigs and shape our analysis of stimulus control. Finally, we discuss potential applications and limitations of our models in medicine and auditory neuroscience.

## Materials and methods

**Figure 1:**
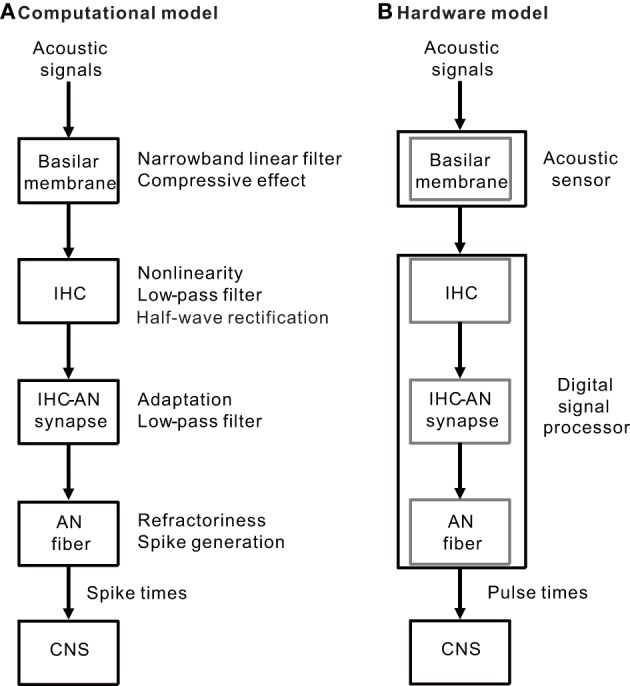
**(A)** A simple block diagram of the peripheral auditory system without feedback processes. The BM of the inner ear functions as narrowband linear filters with a compressive input/output function. The IHC performs functions characterized by non-linearity, low-pass filtering, and half-wave rectification. The IHC-AN synapse includes adaptation and low-pass filtering functions. The AN fiber is a spike generator and has absolute and relative refractoriness. **(B)** Two hardware components (an acoustic sensor and a DSP) were used to implement the auditory peripheral models described in **(A)** The BM was mimicked using a piezoelectric sensor. In addition, a series of the three successive models for the IHCs, IHC-AN synapses, and AN fibers was included in the DSP.

Our model of the peripheral auditory system was built to mimic a series of auditory processes normally carried out in the BM, IHCs, IHC-AN synapses, and AN fibers (Figure 1A). The model was designed to address frequency selectivity, compression, non-linearity, half-wave rectification, low-pass filtering, adaptation, and refractoriness. The model was translated to hardware consisting of two components: an acoustic piezoelectric sensor and a DSP with a pulse-generating function (Figure 1B). The model was not constructed to include all of the functions performed in the auditory periphery, but rather to focus on processes that are essential to auditory processing of a restricted range of sound parameters—i.e., frequencies greater than 1 kHz and SPLs >60 dB. Lower SPLs levels were not examined because active feedback processes that are mainly mediated by outer hair cells (OHCs) were not included in the present model or in any of the currently available CIs. Our goal was to produce AN discharge patterns, and model outputs were primarily compared with previously published AN response properties. When no direct measurements of the parameters were available, the values were set to achieve model responses that most closely simulated the measured responses of AN fibers. Here, we briefly describe the model and associated mathematical function; the details are included in Appendices A to D. Moreover, to discuss the potential applications and limitations of the model, we have included data for electrically evoked auditory brainstem responses (EABRs) obtained from guinea pigs.

### An acoustic sensor to mimic functions of the BM

**Figure 2:**
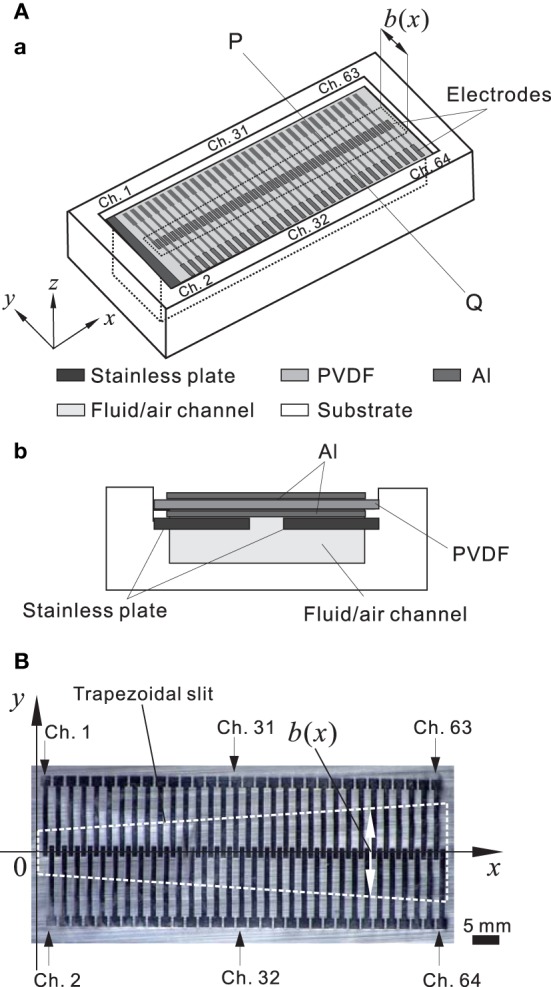
**(A)** Schematic representation of the piezoelectric acoustic sensor. **(A.a)** Three-dimensional view. The three dimensional axes are indicated. The trapezoidal slit shape in the stainless plate is marked with black dashed lines and the width (*b*) of the slit is a function of x, and the origin is the left side of the slit. **(A.b)** Cross-sectional view at the line PQ in **(A.a)**. **(B)** Planer view of a thin PVDF membrane. Sixty-four electrodes were distributed on the membrane. The trapezoidal slit shape in a stainless plate is indicated by white dashed lines.

For the hardware in the model of an artificial BM (ABM), a polyvinylidene difluoride (PVDF) film was used as a piezoelectric sensor to convert sound pressures into corresponding electric signals (Shintaku et al., 2010). A trapezoidal slit was created on a stainless base plate such that the local resonance frequency (LRF) of the membrane over the slit differed based on the position. In the acoustic sensor, electric signals were generated based on the piezoelectric effect from local stress in the membrane. Thus, we obtained frequency selectivity when particular components of a signal were amplified at specific locations on the PVDF film. The piezoelectric acoustic sensor comprises a 30-μm-thick PVDF membrane (Kureha, Japan) bonded to a stainless plate with the narrow slit (Figure 2A) and rectangular (0.54 × 5.0 mm) electrodes distributed along the 31-mm longitudinal axis (Figure 2B). The device was mounted on a substrate with an air-filled (or fluid-filled) channel; the overall size was 47 × 17 and 4 mm deep (Figure 2Ab). In a previous study to represent *in vivo* conditions, the fluid channel was filled with silicone oil (Shintaku et al., 2010). In this study, however, to obtain a resonance frequency range between 1.0 and 20 kHz, we used only an air-filled channel. Because the methods have been previously described in detail (Shintaku et al., 2010), we have only included brief descriptions of differences in the current study. The number of electrodes increased from 24 to 64 channels to obtain a more precise frequency selectivity profile. The 64 electrodes, which are referred to as Ch. 1 to Ch. 64 (Figure 2B), were equally spaced at a distance of 1.0 mm from center to center, resulting in a gap of 0.10 mm between the edges of adjacent electrodes (Figure 2B). A common ground electrode was prepared on the bottom of the ABM. The membrane was then glued on the stainless plate. The size of the trapezoid slit was chosen to obtain the appropriate resonance frequency range (i.e., ~1.0 to 20 kHz). In the slit, the length of the short and long edges was 1.0 and 3.0 cm, respectively. The *x* and *y* axes on the membrane were later defined as shown in Figure 2B. The width *b* of the slit at a position *x* was referred to as *b*(*x*).

We used an experimental setup that was similar to that described by Shintaku et al. (2010). Briefly, a sinusoidal acoustic wave or a harmonic complex sound was applied to the device from one of two speakers (SX-WD, Victor, Japan and FT96H, Fostex, Japan), which was located 50 mm away at a 45° angle. To produce a constant SPL with a precision of ± 0.1 (*SD*) dB at various frequencies, the speaker was previously calibrated using a measuring amplifier (Type 2636, Brüel & Kjær, U.K.). The frequency was controlled between 0.50 and 30 kHz using a function generator (WF 1973, NF Co., Japan) or a frequency response analyzer (FRA 5022, NF Co., Japan). The duration of the sound waveform was between 15 and 50 ms with a SPL between 60 and 100 dB SPL, and a rise/fall time was set to 1% of the stimulus duration. A Hanning window was usually used to obtain a slow onset. The piezoelectric output from the electrodes was measured as a voltage using a custom-made 24-channel preamplifier (NF Co., Japan), which is often referred to as a charge amplifier. For a pure tone in the frequency range between 0.50 and 30 kHz, the steady-state amplitude of the piezoelectric output was recorded and frequency responses were computed. In this study, the bandwidth (*f*_bw_) was defined as the range of frequencies in which the frequency response lay within −10 dB of the response at its peak at a frequency (*f*_0_). Also, the quality-factor *Q* was defined as *Q* = f_0_/f_bw_, which characterized the turning of frequency selectivity at each channel.

In response to characteristic frequency (CF) tones at basal BM sites in the guinea pig, cat, and chinchilla, relationships between SPLs and membrane displacements or volume velocities are compressive between SPLs of 40 and 90 dB (for review, Robles and Ruggero, 2001). Compression of input/output functions is most prominent at moderate and high levels, with average rates of growth as low as 0.2 dB/dB at the CF and even lower rates at frequencies immediately above the CF. Thus, we included this type of compression in our model using a non-linear function to mimic the input/output function. Although the parameters of the function were primarily obtained from previously reported data (Robles and Ruggero, 2001), some parameters were slightly modified for the present study. The details of the function are mathematically described in Appendix A.

### IHC model

For the second step in our model, we addressed the IHCs to convert ABM vibration and the resulting electrical signal output to a signal that corresponded to changes in the membrane voltage. In IHCs, vibration of the stereocilia on the cells opens ion channels and causes a change in the intracellular voltage. Because the IHC membrane has an associated capacitance and a leakage conductance, the IHC membrane low-pass filters the stimulus current resulting from hair-bundle motion. This process can be modeled using a first-order low-pass filter with a 1.1-kHz corner frequency (van Schaik and Meddis, 1999). Thus, together with low-pass filtering by the membrane, asymmetry in the conductance function causes the AC component of the response to decrease with frequency, such that only the DC component remains at frequencies above a few kHz (Palmer and Russell, 1986). For the IHCs, we used a model proposed by van Schaik and Meddis (1999) and modified the original parameters to adjust the electric output of the ABM in our device. The mathematical details are described in Appendix B.

### Model of the IHC-AN synapse

The third step in modeling peripheral auditory processing was to address the IHC-AN synapse, which converts the IHC membrane voltage signal into neurotransmitter release, causing the AN fiber to fire with adaptation. The mechanisms that give rise to synaptic adaptation at the IHC-AN synapse include depletion of a primed presynaptic pool of neurotransmitter (Moser and Beutner, 2000) and desensitization of postsynaptic receptors (Raman et al., 1994). The adaptation at the IHC-AN synapse has often been modeled using multiple neurotransmitter reservoirs, including diffusion out of the cell and between reservoirs within the cell (Furukawa and Matsuura, 1978; Schwid and Geisler, 1982; Smith and Brachman, 1982; Meddis, 1986a; Westerman and Smith, 1988; Ross, 1996). During the past several decades, two major types of IHC-AN synapse models have been developed independently in a series of studies by two research groups (Meddis, 1986b; Westerman and Smith, 1988; Meddis et al., 1990; Carney, 1993; Zhang et al., 2001; Sumner et al., 2002). Zhang and Carney, however, have recently reported that the two model types are essentially the same despite their different structures (Zhang and Carney, 2005). In addition, both model types were based on the observation that synaptic adaptation to the onset of AN firing in response to tone bursts is usually characterized by two major exponential components and potentially one or more additional minor exponential component(s). Thus, to mathematically describe the onset of AN responses to tone bursts, two or more independent variables are likely needed; the variables are governed in parallel by first-order systems. Based on these two model types, we propose a simple framework for a model of the IHC-AN synapse. The output of the AN model represents the instantaneous AN synapse signal *s*, and was modeled as

(1a) $$dx/dt=P(t)x+p_{0},$$

(1b) s=K(t)x+σξ(t),

where ***x*** represents concentrations in the multiple reservoirs, and *p*_0_ is a steady-state input that causes spontaneous activity in the AN fiber (see Appendix C). Here, although the matrix *P*(*t*) and the vector *K*(*t*) include time-varying inputs to the AN fiber, the elements can be constant for a sinusoidal input at higher frequencies. Thus, under these conditions, Equation (1a) is a first-order system with the multivariable ***x*** and is characterized by major exponential components of the coefficient matrix *P*. Additionally, in Equation (1b), σ is an observed noise intensity and ξ(*t*) is the standard Gaussian white noise.

Connecting the IHC-AN synapse model to the IHC model and the ABM device model required multichannel components. Therefore, we extended the single-channel IHC-AN synapse model to a multichannel version by assuming that each channel in the IHC-AN synapse model was independent and the model structure was identical, whereas each set of parameters was different. For previously reported experimental data (e.g., Westerman and Smith, 1984), the parameters in the onset adaptation function (Equation C8 in Appendix C) were usually determined by fitting a characteristic equation to the poststimulus time histogram (PSTH) of AN fiber responses. In studies of cat primary-like AN fibers, neural activity quickly rose to a peak followed by rapid adaptation with a time constant of 1–15 ms (Harris and Dallos, 1979; Smith and Brachman, 1980). Thereafter, the activity decreased more slowly, and the adaptation time constant ranged from 15 ms to more than the duration of the evoked stimulation (e.g., 30–60 ms). Furthermore, in Mongolian gerbils, the two major time constants for adaptation in primary-like AN fibers depended on the CFs, and the rapid adaptation time constant decreased with an increasing CF (Westerman and Smith, 1984). The CF-dependent properties of the two major time constants for other species, however, have not yet been reported. Therefore, we assumed three points for the model in this study: (i) LRFs for the piezoelectric membrane correspond to the CFs in the AN model, (ii) the rapid and slow adaptation time constants are CF-dependent, and (iii) the relationships between the time constants and CFs can be described using a logarithmic function (see Appendix C). Based on assumption (i), the LRFs were replaced with CFs in the AN model. Although the parameters were partly based on data reported by Westerman and Smith (1984), this assumption required further investigation. Several subtypes of AN fibers, including fibers that are characterized by high, medium, and low spontaneous firing rates, have been described. In this study, however, we limited the analysis to fibers with high spontaneous firing rates.

### A discharge generator to model an AN fiber

The last step in modeling peripheral auditory processing was to incorporate a spike discharge generator as an AN fiber model. The model discharge times were generated using a non-homogenous Poisson process driven by the synaptic signal *s*(*t*) of the IHC-AN synapse model. The discharge generator model was basically the same as that proposed by Zhang et al. (2001). Like their model, our model included the refractory effects of AN fibers. The details of the model are described in Appendix D.

### Model implementation in a DSP and data acquisition

All simulations using the IHC, IHC-AN synapse, and AN fiber models were carried out using a DSP (iBIS DSP7101A, MTT Co., Japan), which had 16 input and 8 output analog channels. For the simulations, each component of the model was divided into 64 sections. The distribution of CFs, which corresponded to the LRF in the ABM, was set along the frequency axis using data obtained from humans or cats (Liberman, 1982; Greenwood, 1990, 1991). The parameters values for our simulations are reported in Appendix A to E. In the DSP, the differential equations were solved using the Runge–Kutta fourth/fifth-order method. A time step of 5 μ s was used for all numeric calculations. The output of the models was sampled at 200 kHz using an NI PCI-6259 DAQ card (National Instruments, Austin, TX, USA) and custom data acquisition software written in MATLAB R2012 (Mathworks, Natick, MA, USA).

### Experimental animals

A total of six male Hartley guinea pigs (4–10 weeks old, 300–600 g; Japan SLC, Japan) with a normal pinna reflex served as the experimental animals. All animals had normal hearing based on auditory brainstem responses to sound clicks. Animals were cared for under the supervision of the Animal Care and Use Committee at Hokkaido University (Japan). All experimental procedures followed the National Institutes of Health Guidelines for the Care and Use of Laboratory Animals. When general anesthesia was required, the animals were administered an intramuscular injection of 10 mg/kg midazolam (Astellas Pharma, Japan) and 0.01 mg/kg xylazine (Bayer AG, Germany) in normal Ringer's solution. Midazolam has been reported to specifically target γ-aminobutyric acid A (GABA_A_) receptors and modify inhibitory postsynaptic currents in the central nervous system. The dose of midazolam used in this study, however, does not affect these spontaneous and evoked inhibitory postsynaptic currents (Verbny et al., 2005). Supplemental doses were administered every 1 h or more often if the animal withdrew its leg in response to applied pressure. During the experiment, body temperature was maintained at 37 ± 1°C using a heating pad.

### Electrode implantation and EABR recordings

Measurements of electrically EABRs were performed as described in previous studies (Hall, 1990b; Ogita et al., 2009; Shintaku et al., 2013). Briefly, we used a conventional retroauricular approach with the animal in a lateral recumbent position. After exposing the otic bulla, a small hole was made in the otic bulla to expose the round window niche and the basal turn of the cochlea. One platinum/iridium electrode (200-μm diameter) was inserted into the scala tympani through the round window and placed approximately 1.5 mm deep in the basal portion of the cochlea. Another electrode was fixed to the temporal bone where it served as an extracochlear ground. Voltage pulses were generated using a computer and a real-time processor (RP2.1, Tucker-Davis Technologies, USA) and the voltage pulses were used as a command signal for current stimulators (DS 8000, WPI, USA or SIU-91, Cygnus Technology, USA). Stimulus current levels were calibrated by measuring the voltage with an oscilloscope. The responses to electrical stimuli were recorded using stainless steel needle electrodes (positive, vertex; negative, neck; ground, thorax). The electrical stimulus consisted of monophasic current pulses (0.6-ms pulse width) occurring 50 times per second. Positive or negative current pulses were alternatively applied to the electrode in a single-pulse or double-pulse mode (see Results). The response signals were digitally amplified, band-pass-filtered in a frequency range of 200–1000 Hz, sampled at 25 kHz, and averaged over 500 trials using response-processing equipment (RA16 and PR2.1, Tucker-Davis Technologies).

### Data analysis

Results obtained with the model are shown as averaged data points, because there was little variance in the data. In contrast, results obtained during the EABR recordings are shown with error bars representing standard errors. Using unpaired *t*-tests, *P* values less than 0.01 were considered statistically significant. Data are presented as means ± standard errors, when necessary.

## Results

### Responses of the piezoelectric sensor

**Figure 3:**
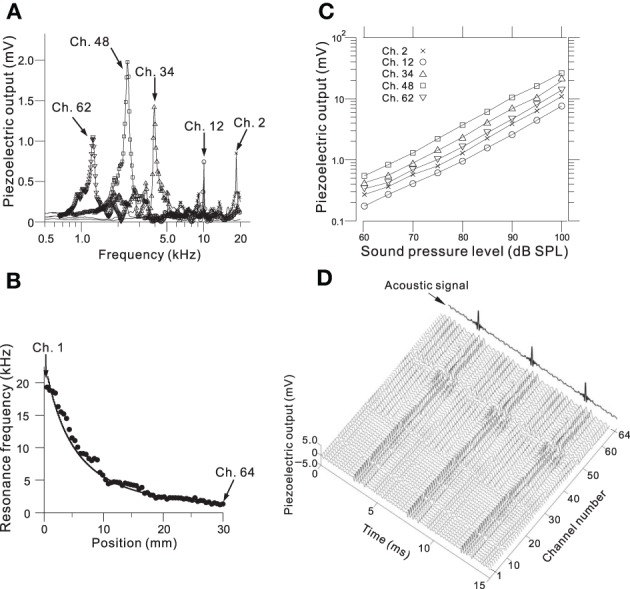
**(A)** Frequency characteristics of the amplitude of the piezoelectric voltage output for Ch. 2, 12, 34, 48, and 62. Frequencies of the input sound ranged from 0.5 to 20 kHz with a step size of 20 or 50 Hz, and the amplitude of the SPL was 75 dB. **(B)** Relationship between position *x* in Figure 2B and the LRF for all 64 channels. The circles indicate the observed LRFs, and the curve obtained from numerical calculations in the theoretical analysis have been superimposed (see Appendix A). Ch. 1 and 64 are indicated by arrows, and the channel number increases from left to right. The input sound was the same as described in part A. **(C)** Relationship between SPLs and piezoelectric output amplitudes for Ch. 2, 12, 34, 48, and 62 for SPLs from 60 to 100 dB with a step size of 5 dB. **(D)** Piezoelectric output waveforms for all 64 channels in response to an acoustic signal containing a 210-Hz harmonic complex sound (indicated by an arrow).

We determined the steady-state peak amplitudes of the voltage output from the piezoelectric sensor in response to pure tones with an SPL of 75 dB and various frequencies at five channels on the ABM (Figure 3A; Ch. 2, 12, 34, 48, and 62). For each channel, we observed one major peak and several additional sideband peaks, which indicated that the ABM had at least one major LRF at each channel. The amplitudes of the piezoelectric outputs at channels with higher LRFs (e.g., Ch. 2 and 12 in Figure 3A) and lower LRFs (e.g., Ch. 62) were smaller than that at channels with LRFs in the middle range (e.g., Ch. 34 and 48). In addition, the LRF of each channel differed (Figure 3B), ranging from higher to lower frequencies from Ch. 1 to Ch. 64. In the lower frequency range (1–7 kHz, Ch. 24–64), the measured LRFs (circles in Figure 3B) agreed with values calculated using a theoretical analysis (thick curve; see Appendix A). In the higher frequency range (7–20 kHz, Ch. 1–23), however, a small discrepancy between the measured and calculated LRSs was observed. Figure 3C shows the relationships between SPLs and the corresponding voltage outputs from the piezoelectric sensor. These relationships were approximately linear, suggesting that the piezoelectric device can be used as a linear system to detect SPLs with frequency selectivity. These results are similar to those reported in previous studies (Shintaku et al., 2010; Inaoka et al., 2011). Some of the resonance properties—i.e., LRF, bandwidth, and Q-factor—for the five channels are listed in Table 1. Figure 3D depicts the responses of all 64 channels of the ABM when a 210-Hz harmonic complex signal was used as the input stimulus (labeled “Acoustic signal” in the figure). The lower-order harmonics were resolved for the low-frequency sections of channels with the higher channel numbers (e.g., Ch. 50–60). In contrast, higher-order harmonics were represented in channels with the lower channel numbers that displayed amplitude modulation (c.f., Figure 7).

**Table 1:** **Summary of resonance properties in five channels**.

**Channel No.**	**Local resonance frequency, *f_0_* (kHz)**	**Bandwidth, *f_bw_* (kHz)**	**Q-factor, *Q* = f_0_ /*f_bw_***
2	18.6	2.17	8.57
12	10.1	0.820	12.3
34	4.25	0.490	8.67
48	2.39	0.407	5.87
62	1.24	0.225	5.51

### Responses of the auditory peripheral model

**Figure 4:**
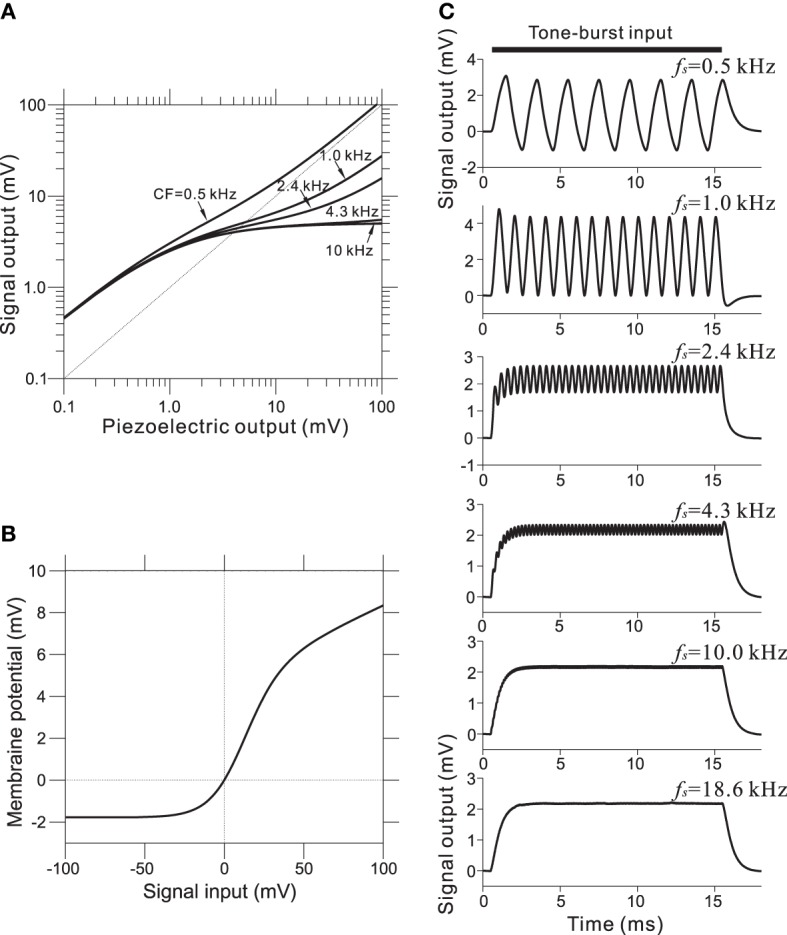
**(A)** Compressive effects of the BM were mimicked using CF-dependent non-linear functions. Each relationship depends on the corresponding CF of the BM site. **(B)** Asymmetrical relationships between input signals (compressive output of the piezoelectric sensor) and the output signal for the IHC model. **(C)** Intracellular voltage from the IHC model in response to pure-tone bursts. The duration of each pure-tone burst was 15 ms, as indicated by a thick line on the top of graphs, and the amplitude of stimulation was 4.0 mV. The LRF (*f*_s_) of the piezoelectric membrane where each model IHC was located is indicated on the upper right of the corresponding trace of the model waveforms.

To incorporate the compressive effect observed in the BM of the cochlea, non-linear relationships of this property were directly modeled using a non-linear transformation between the piezoelectric signal and the corresponding voltage output. The compression of input/output functions is most prominent for middle and higher SPLs and for higher CF tone bursts; the CF-dependent properties of the model are illustrated in Figure 4A (for details, see Appendix A). Furthermore, asymmetry in the conductance in IHCs was modeled using a simple asymmetric non-linear function (Figure 4B and Appendix B). The model was based on reported data (Russell et al., 1986), and the range of input variables was adjusted for our model. In the IHC model, low-pass filtering caused the AC component of the response to decrease with frequency in response to sinusoidal inputs between 0.5 and 5 kHz (Figure 4C). Furthermore, only the DC component remained at input frequencies greater than 5 kHz.

**Figure 5:**
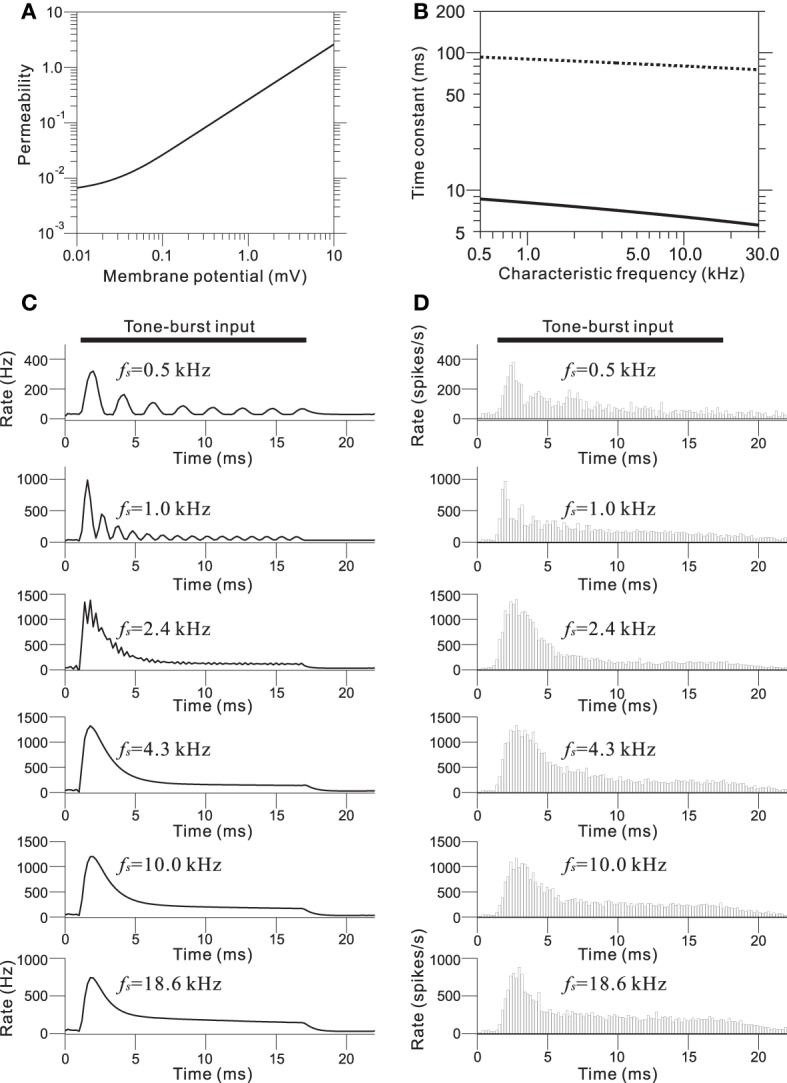
**(A)** Transmitter permeability as a function of membrane potential in the model of the AN fiber. **(B)** Two time constants of the AN fiber model as a function of the CF of the fiber. **(C)** Output waveforms of the IHC-AN synapse model. The LRF (*f*_s_) of the piezoelectric membrane where each model IHC-AN synapse was located is indicated on the upper left of the corresponding trace of the model waveforms. **(D)** PSTHs based on 500 trials and a 0.2-ms bin size. The LRF (*f*_s_) of the piezoelectric membrane where each model AN fiber was located is indicated on the upper left of the corresponding trace of the model waveforms.

In the IHC-AN synapse model, the output from the IHC model was transformed into an immediate permeability (see Materials and Methods and Appendix C). The relationship between the permeability and the membrane potential from the IHC model was a soft non-linear function (Figure 5A). The output synaptic activity was modeled using a second order system with fast and slow time constants (Figure 5B). The parameter values for the time constants were CF-dependent and decreased with the CF. Figure 5C shows representations of the synaptic activity from the IHC-AN synapse model; the input signals are illustrated in Figure 4C. Models of IHC-AN synapses characterized by CFs <2 kHz resulted in synaptic activity waveforms that were phase-locked in response to the corresponding IHC output signals. In contrast, models of the IHC-AN synapse with higher CFs (>2 kHz) produced synaptic activity waveforms that did not follow each cycle of the sinusoidal input to the IHC model. In response to signals from the IHC model, we observed a relative increase during the onset period (<2 ms), which was a result of the adaptation included in the IHC-AN synapse model. This adaptation can also be illustrated using PSTHs of the responses to the same inputs, although stochastic properties blurred the rapid transients (Figure 5D). In addition, the shape of the PSTH slightly changed as the SPL increased (data not shown) because of the relative and absolute refractoriness of the spike generator (Appendix D).

**Figure 6:**
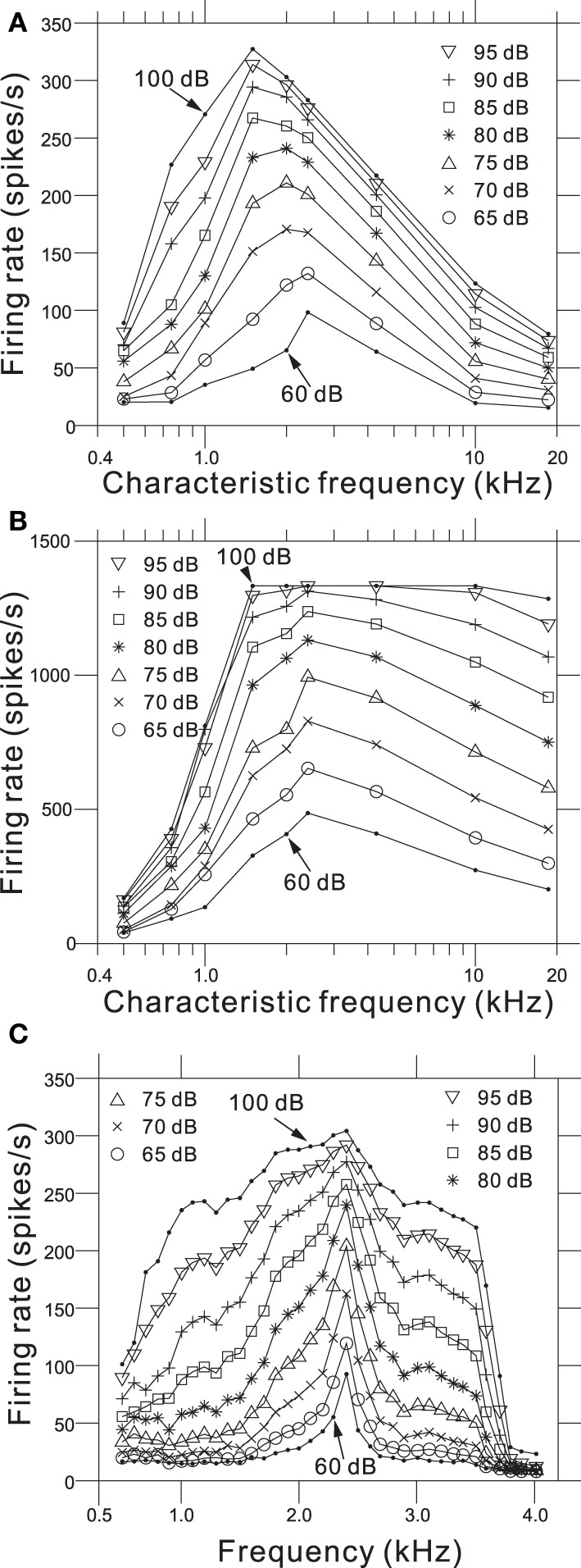
**(A)** Steady-state firing rate as a function of CFs for 9 AN fiber models in response to pure tones with nine different SPLs. **(B)** Maximum firing rate of onset transients as a function of CFs for the same AN fiber models. **(C)** Intensity-dependent discharge rate for a model fiber with a CF of 2.39 kHz. Rates were computed from the sustained responses to 500 trials with a 50-ms pure-tone burst. The model fiber corresponds to Ch. 48 on the piezoelectric membrane.

We determined relationships between the intensity-dependent firing rates and the CFs using the steady-state firing rates (Figure 6A) and the transient peaks in the firing rates (Figure 6B). The model showed the best frequency sensitivity in the range between 1 and 2 kHz (Figure 6A). For steady-state firing rates in response to pure tones, the maximum firing rate was CF-dependent and shifted to lower frequencies as the SPL increased. In addition, the corner frequencies of the transient peaks also changed in an SPL-dependent manner (Figure 6B). Firing rate responses to pure tones at frequencies away from the CF provide information about the non-linear tuning of the model. Figure 6C illustrates the response area for the output of one AN fiber model with a CF of 2.39 kHz, and changes in the firing rate as a function of SPL for frequencies greater and less than the CF. The specific channel (Ch. 48 with an LRF of 2.39 kHz) was compared with the example reported in Figure 8 from Anderson et al. (1971). The response area—i.e., isolevel contours of the firing rate—spread as the input level increased, which was similar to the previous result (Anderson et al., 1971). An SPL-dependent shift in the peak frequency of the response area was not observed in the results owing to approximate symmetry of the piezoelectric membrane around the LRF, which revealed a limitation of our model. In addition, for larger SPLs (>85 dB), steep decreases were not observed greater than the CF, because second- or higher-order resonance frequencies influenced the side-band properties, suggesting a second limitation of the model.

**Figure 7:**
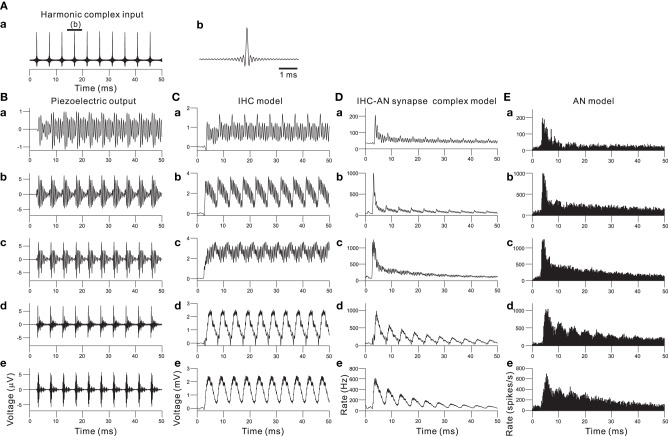
**(A)** Input signal of a 210-Hz harmonic complex sound. The peak SBL was 95 dB. **(A.b)** shows an expanded view of the input signal for the interval indicated by the thick bar on the top of **(A.a)**. **(B)** In response to the harmonic complex input, piezoelectric output waveforms from the acoustic sensors at Ch. 62, 48, 34, 12, and 2 are shown in **(B.a)**, **(B.b)**, **(B.c)**, **(B.d)**, and **(B.e)**, respectively. For Ch. 2, 12, 34, 48, and 62, the CFs are 1.0, 2.4, 4.3, 10.0, and 18.6 kHz, respectively. **(C)** Output waveforms from the IHC model. **(C.a)**, **(C.b)**, **(C.c)**, **(C.d)**, and **(C.e)** correspond to the description in **(B)**; lower frequency to higher frequency sections are shown from the top to the bottom. **(D)** Responses of the IHC-AN synapse model. The arrangement of the sections is the same as described for **(C)**. **(E)** Responses of the AN fiber model. The arrangement of the sections is the same as described for **(C)** and **(D)**.

Combining the piezoelectric sensor and the auditory peripheral model, including the IHC, IHC-AN synapse, and AN fiber models, resulted in the responses shown in Figure 7 when a 210-Hz harmonic complex signal was applied as the acoustic input (Figure 7A). The responses of the piezoelectric sensor showed that first lower-order harmonics were represented in the low-frequency sections (Figure 7B). In contrast, higher-order harmonics were represented in the rest of the sections. The responses displayed much larger amplitude modulation and characteristic wave packets as the section number decreased (i.e., higher frequency sections). In Figure 7C, the output of the IHC model shows a rectification effect and some distortion due to the non-linearity demonstrated in Figure 4B. In higher frequency sections (e.g., Ch. 2 and 12 in Figure 4C), the AC component of the output decreased, and the envelope of the wave packet was mainly represented by the output of the IHC model. Thus, each cycle of the increased transient in the envelope was phase locked to that of the input signal of complex harmonics (Figure 7A). The next step was to model neurotransmitter release and the resulting adaptation. The adaptation caused the AN response to be strongest at the onset of a sound and then decrease to a steady state if the input was periodic. The output of the IHC-AN synapse model showed adaptation responses to complex harmonics (Figure 7D). The final stage was to add the AN fiber model. Responses from the model were converted to PSTHs using repeated simulations in 500 trials with the complex harmonics (Figure 7E). The results obtained with the AN fiber model were similar to the output of the IHC-AN synapse model, although jitters in the spikes affected the output timing.

### EABRs evoked by single- and double-pulse stimuli

**Figure 8:**
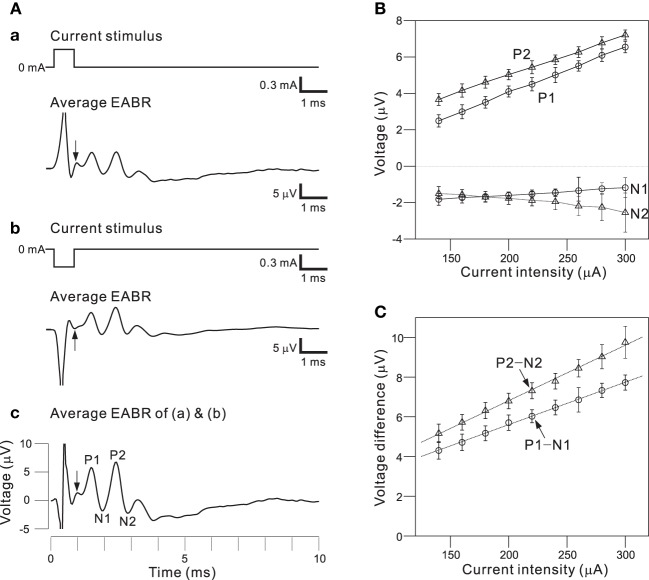
**(A)** Averaged EABRs induced by 0.6-ms **(A.a)** positive (300 μA) current pulses and **(A.b)** negative (−300 μA) current pulses. Each trace represents an average of 500 responses. A trace representing the average of all 1000 responses is shown in **(A.c)**. In the waveform, positive and negative peak amplitudes are sequentially labeled P1, N1, P2, and N2. The offset times of the stimuli are indicated by arrows. **(B)** Relationships between peak amplitudes (P1, N1, P2, and N2) and intensities of current pulses. **(C)** Relationships between peak-to-peak amplitudes (P1-N1 and P2-N2) and intensities of current pulses. Regression lines for the peak-to-peak amplitudes are denoted with dotted lines.

To use the auditory peripheral model in CIs, we must understand the final transformation of electrical stimulations to neural discharges. Therefore, we examined the effects of a short pulse and two successive pulses on activity in a population of AN fibers; pulse intensities and interpulse intervals (IPIs) were controlled as stimulation parameters. To minimize electrical artifacts induced by stimulation, positive and negative pulses were alternatively applied in the same trial and evoked responses were averaged (Figures 8Aa,b). For the average EABR, two positive and negative peaks were clearly identified, and labeled P1, N1, P2, and N2 beginning with the peak closest to the onset (Figure 8Ac). Generally recognized as the compound action potential of the AN, first positive wave in the EABR should reflect the number of excitable AN fibers (Goldstein and Kiang, 1958; Simmons and Smith, 1983; Hall, 1990a). In addition, P1–N1 growth functions can serve as an approximate measure of AN fiber activity (Hall, 1990a). Therefore, we calculated two measures of the response amplitudes: (i) the peak amplitude (P1 and P2), the difference between the prestimulus baseline and the peak; and (ii) the peak-to-peak amplitude (P1-N1 and P2-N2), the difference between the peak and the following trough.

Figure 8B shows the relationships between local maximum and minimum peaks (P1, P2, N1, and N2) and the pulse intensities. The amplitudes of P1, P2, and N2 were proportional to the current pulse intensities, whereas the N1 amplitudes were nearly constant for various applied current intensities. Because absolute values of peak amplitudes were easily influenced by noise and the values differ from sample to sample, we also examined peak-to-peak amplitudes (e.g., P1-N1; Figure 8C). The relationships between the peak-to-peak amplitudes and the pulse intensities were approximately linear in the range of tested intensities. Thus, the P1-N1 amplitude should be directly proportional to the number of excitable AN fibers.

**Figure 9:**
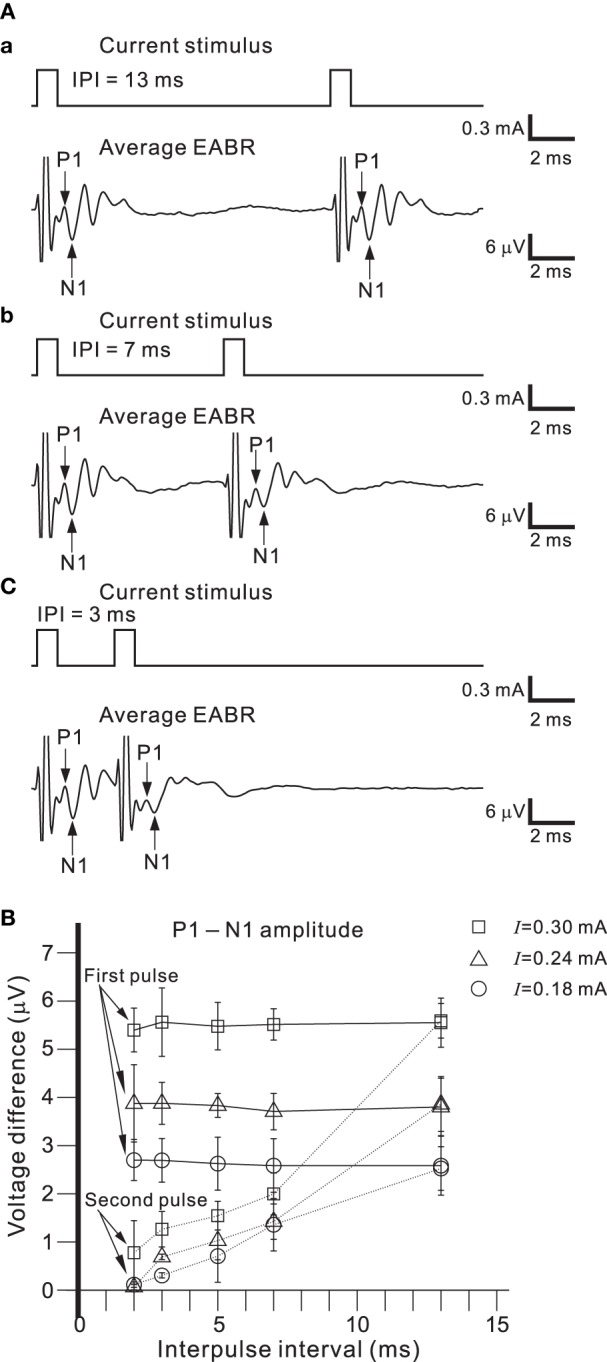
**(A)** Averaged EABRs induced by double current pulses with a duration of 0.6 ms and an intensity of 300 μA. The IPI was 13, 7, and 3 ms in **(A.a)**, **(A.b)**, and **(A.c)**, respectively. Although positive and negative pulse stimuli were alternatively applied in the experiment, only the results for a positive pulse stimulus are illustrated. **(B)** Relationships between peak-to-peak amplitudes (P1-N1) and IPIs for three levels of current pulse intensities. The intensities were 0.18, 0.24, and 0.30 mA, and are denoted by circles, triangles, and squares, respectively.

Figure 9A shows EABRs from part of series of IPIs. If the IPI was >10 ms, the difference between the P1-N1 amplitudes of the EABRs produced by the first and second pulses was not significant (Figure 9B; *p* > 0.01). In contrast, if the IPI was <7 ms, P1-N1 amplitudes of the EABRs produced by the second pulses were significantly lower than those produced by the first pulses (*p* < 0.01). Consequently, the characteristic responses of the AN population were markedly affected by the temporal pattern of the series of single pulse trains.

## Discussion

### Overview of the model of the peripheral auditory system

In this study, to mimic AN responses in the peripheral auditory system, we have described a simple model comprising an ABM and models of IHCs, IHC-AN synapses, and AN fibers. For the ABM, we microfabricated a PVDF membrane with 64 electrodes and a ground. We used a piezoelectric sensor to convert sound pressure and the resulting mechanical displacement into electric signals. To model frequency selectivity on the ABM, a slit was created on a stainless base plate to obtain overlying membrane LRFs, which correspond to CFs in a real BM. Electric signals were generated owing to the piezoelectric effect from local stress in the membrane. Thus, the electrode at the resonating location produced a relatively large output signal, and frequency selectivity was attained using the resonance of vibration and the electrode array. In addition, computer models that included non-linear and asymmetric input/output functions were sequentially connected to the sensor. The responses of the overall model to pure-tone burst and complex stimuli resulted in reasonably accurate representations of the discharge rates of AN fibers at frequencies greater than 1 kHz with middle to high SPLs.

### Frequency selectivity

CIs must be constructed to recreate the frequency selectivity observed in the cochlea. The perception of speech would be markedly hindered by a spectral mismatch between the population of AN fibers excited by a given electrode and the frequency range of the sound presented to that electrode or by distortions in the speech processors of CIs (Bernstein et al., 2013; Grant et al., 2013). Moreover, frequency selectivity is not only mediated by the auditory periphery. In addition to cochlear neurons, cells in other brain areas, including the brainstem, inferior colliculus, thalamus, and auditory cortex, contribute to the frequency selectivity (Romand, 1997; Froemke and Jones, 2011; Hackett et al., 2011). In various cortical areas, the neurons encode receptive fields that cover a range of sound frequencies (Ojima et al., 2005; Tsytsarev et al., 2009). These neurons also contribute to speech perception.

### ABM structures in previous models

Two primary approaches have been used in ABM mechanics. One type of artificial cochlea includes devices with fluid-filled 1:1 scale models of the human cochlea that respond appropriately to sound signals (Grosh and White, 2005; White et al., 2008). Although these devices were built using micromachining technology to allow the integration of new sensory elements, current devices are primarily used for research into the mechanics of the cochlea. Thus, new types of CIs or neural prostheses are desired. A second type of artificial cochlea includes a mechanical bank of resonators that was designed to respond like the human cochlea. In these devices, the mechanical bank of resonators passively sub-band filters the signals. To create the filter bank, for example, Haronian and MacDonald employed a large array of thin bridges micromachined in silicon with lengths that increased exponentially (Haronian and MacDonald, 1996). The result was an array of resonators, each with a specific CF, although the Q factors of the resonators were lower than those recorded in the cochleae of rodents (*Q* = 5−10), cats (*Q* ≈ 11), and squirrel monkeys (*Q* ≈ 5) (Robles and Ruggero, 2001). Thus, the Q factors of the ABM in the present study were appropriate for models of these animals (c.f., Table 1). In addition, Tanaka et al. fabricated a “fish bone” resonator device, consisting of an array of mechanical beams connected to a single central torsional beam (Tanaka et al., 1998). This device mimicked a cochlea, behaving as an acoustic transmission line, although external optical instrumentation was needed to monitor the movements of the resonators.

Furthermore, a “silicon cochlea” refers to electronic circuitry that is designed to convert sound signals into multiple outputs (Lyon and Mead, 1998; van Schaik and Meddis, 1999; Mandal et al., 2009; Wen and Boahen, 2009). Lyon and Mead described the first silicon cochlea, opening up the field of neuromorphic engineering (Lyon and Mead, 1998). This research led to devices containing banks of band-pass filters with an electronic transmission line (filter cascade). Using the cascading series of filters, a large number of outputs can closely model the gain, filtering, and dynamic range characteristics of the cochlea (Lyon and Mead, 1998). Furthermore, recent advances have resulted in similar devices that are small (<3 × 3 mm) and consume little power (e.g., 0.5 mW) (Sarpeshkar and Lyon, 1998; Sarpeshkar, 2010). Although these approaches differ based on fluidic, mechanical, and/or electrical parameters, developing a small, low-power, analog, multiresonator system that can mimic the cochlea is likely to be a major step toward a completely bionic implantable ear.

### Structure of our ABM model

The ABM output amplitude, frequency tuning, and range of LRFs depended on several parameters in the model and device—e.g., width of the slit, thickness of the membrane, and membrane materials. Further optimization of these parameters will be needed to incorporate the model into a usable neural prosthesis. In particular, the choice of the piezoelectric membrane provides the important benefit of no power consumption during the transformation of acoustic signals to electric signals. In general, several substances exhibit piezoelectricity, including natural crystal (e.g., quartz and rock crystal), man-made crystal (e.g., gallium orthophosphate and langasite), natural ceramics (e.g., lead zirconate titanate and lead zirconium titanate), man-made ceramics (e.g., lithium niobate), and polymers (e.g., PVDF). Among these materials, the piezoelectricity of PVDF is several times greater than that of quartz. Additionally, compared with other fluoropolymers, PVDF has a relatively low melting point at approximately 177°C (Tashiro et al., 1983). Despite the advantages of PVDF as a thin film for microfabrication processes, future work should continue to search for better materials to generate larger output signals and increase the transformation efficacy.

### Non-linear properties of computational models

The active process of the BM is affected by a wide region of this structural element of the cochlea (Delgutte, 1990; Zhao and Santos-Sacchi, 1999). Moreover, tones at frequencies greater and less than the CF have different effects on non-linear tuning (Delgutte, 1990; Ruggero et al., 1992). Experimental data from IHCs and AN tuning curves also suggest that OHCs basal to the IHCs are responsible for enhancing the vibration of IHCs (Patuzzi and Yates, 1986). In many studies, OHCs have been proposed to play an important role in a “feedback” mechanism that contributes to cochlear non-linearity (review for, Patuzzi and Robertson, 1988; Robles and Ruggero, 2001). In some computer models, saturating non-linearity and a feedback path of low-pass filters represent mechanoelectrical transduction and low-pass filtering owing to properties of the OHC membrane (Robert and Eriksson, 1999; Zhang et al., 2001). Moreover, filtering by the BM was simulated using a non-linear feedback path to control the bandwidth of the narrow-band filter (Giguere and Woodland, 1994; Robert and Eriksson, 1999; Zhang et al., 2001). We, however, believe that more data is required to precisely characterize feedback non-linearity and gain control in OHCs. Furthermore, because gain control in OHCs is effective at low sound intensities and the objective of this study was limited to middle to high sound intensities, any potential feedback path would not be examined in the present model. In addition, the lack of gain control in the feedback path comes with the practical benefit of inherently stable model dynamics if the model is used in CIs or other neural prostheses. The passive nature of the model allows a great deal of freedom in the choice of parameters (e.g., IHC-AN synapse model and Appendix C), which may enable it to reflect AN responses over a relative large range of CFs and SPLs. The flexibility in the choice of parameter values can make it easier to match AN responses in specific device types and animal species.

### An fiber responses and other types of AN fibers

Our model responses to pure-tone burst and complex stimuli provide reasonably accurate representations of the discharge rates of AN fibers characterized by high spontaneous rates at frequencies greater than 1 kHz and middle to high SPLs. Several AN response properties, however, were not included in this model, which should be addressed in future studies. For instance, the model does not include the tails of tuning curves (Kiang and Moxon, 1974; Liberman and Kiang, 1978), representing the glide in the instantaneous frequency of the AN impulse response that is related to the level-dependent shift in the best frequency (Carney et al., 1999). Additionally, our model did not include the effects of middle- and external-ear acoustics (Rosowski and Merchant, 1995; Ravicz et al., 1996) or the effects of efferent nerves on the rate and timing of AN discharges (Guinan and Stankovic, 1996). In particular, because time-related phase modulation is clearly important, we are presently studying the phases of the responses. In addition, the model should be able to simulate responses over a wide range of CFs to allow additional studies of AN models with different spontaneous rates. Therefore, we need to extend this model to include fibers with low or medium spontaneous firing rates (Liberman, 1978) to accurately describe AN responses and examine their role in encoding pure tones and complex sounds.

### Stimulus control and EABR responses

Better replication of the processing that occurs in the auditory periphery will depend on (i) the use of appropriate models and (ii) a requisite level of stimulus control. The latter is limited in currently available implant systems, but may improve in the future. Even in response to stimulation with the same pulse rate (e.g., 2 pulses per 10 ms in Figure 9), the EABRs were considerably different owing to the IPIs. Improvements in stimulus control may allow a closer approximation of the normal patterns of discharge in the AN using an auditory prosthesis and electrical stimuli. Therefore, how to encode an AN firing rate into stimulus-pulse timings in response to a natural sound is an important point for stimulus control. A closer approximation of AN responses would require accurate auditory models that reproduce all important details of processing peripheral to the AN and perhaps even efferent control of the cochlea and the stapedius muscle. Compared with simpler models, however, using sophisticated models in conjunction with limited or poor stimulus control may actually degrade overall performance. Thus, a balance should be sought between model complexity and the level of stimulus control. Models of the auditory periphery, including appropriate transformations from AN responses to stimulus-pulse trains, will be necessary to create better neural prostheses.

## Conclusions

We have modeled the auditory periphery by combining a piezoelectric acoustic sensor and a computer model that has been introduced into a DSP. In response to acoustic stimuli greater than 1 kHz and SPLs greater than 60 dB, the model produced reasonable representations of the activities of AN fibers with high spontaneous firing rates. Our data on electrically EABRs in guinea pigs also highlighted the importance of stimulus control. As a whole, our approach will support the development of new CIs and neural prostheses and will provide a tool for investigations into information processing in the peripheral auditory system.

## Conflict of interest statement

The authors declare that the research was conducted in the absence of any commercial or financial relationships that could be construed as a potential conflict of interest.

## Abbreviations

ABM: artificial basilar membrane
AN: auditory nerve
BAS: bionic acoustic sensor
BM: basilar membrane
CF: characteristic frequency
CI: cochlear implant
DSP: digital signal processor
EABR: evoked auditory brainstem response
IHC: inner hair cell
IPI: interpulse interval
LRF: local resonance frequency
OHC: outer hair cell
PSTH: poststimulus time histogram
PVDF: polyvinylidene difluoride
SPL: sound pressure level
