# Supplementary material for: A hardware model of the auditory periphery to transduce acoustic signals into neural activity
Source: Front Neuroeng. 2013 Nov 26;6:12. doi: 10.3389/fneng.2013.00012 (PMC3840400; doi:10.3389/fneng.2013.00012)
Supplement: Supplementary file 1 [file DataSheet1.PDF]

Appendix A

Cochlear Model. A three-dimensional cochlear model and the structure of the corresponding ABM were previously described (Steele and Taber, 1979; Shintaku et al., 2010). In the model, the governing equation for the bending vibration of a plate (i.e., the BM) with isotropic mechanical properties can be described as

$$\rho_m h\partial^2 w/\partial t^2 + D[\partial^4 w/\partial x^4 + 2\partial^4 w/\partial x^2\partial y^2 + \partial^4 w/\partial y^4] = p_0, \quad \text{(A1)}$$

where D, p_0, w, and ρ_m are the bending rigidity, pressure of the acoustic wave, displacement in the z direction, and density of the BM, respectively. The fluid/air is assumed to be incompressible and inviscid, and the flow is governed by Laplace's equation: $\nabla^2\varphi_\mathrm{f} = 0$, where φ_f is the velocity potential of the fluid/air in the x, y, and z directions, and defined by $\dot{u} = \partial\varphi_\mathrm{f}/\partial x$, $\dot{v} = \partial\varphi_\mathrm{f}/\partial y$, and $\dot{w} = \partial\varphi_\mathrm{f}/\partial z$. The displacement components are u, v, and w, and the dots denote differentiation with respect to time. The subscript f in the velocity potential is u or l, which indicates fluid/air at the upper or lower surfaces of the BM, respectively. The velocity potential φ_f is related to the pressure based on $\rho_\mathrm{f}\partial\varphi_\mathrm{f}/\partial t = -p_\mathrm{f}$, where ρ_f is the density. The governing equations were solved using Dirichlet boundary conditions at the walls of fluid channels: $\dot{v} = \partial\varphi_\mathrm{f}/\partial y = 0$ at $y = \pm L_1/2$ and $\dot{w} = \partial\varphi_\mathrm{l}/\partial z = 0$ at $y = -L_2$, where L_1 and L_2 are the width and depth of the fluid channel, respectively. In addition, the kinematic boundary condition at $z = 0$ is written as $\partial w/\partial t = \partial\varphi_\mathrm{f}/\partial z$. The pressure p_0 is the pressure difference between the top and bottom of the BM and can be approximated by $p_0 \cong -\rho_1\partial\varphi_1/\partial t$ at $z = 0$. To obtain an oscillatory solution of w at the periodic steady state, a single mode $\eta(x, y)$ was assumed for the shape function of the BM and determined from the analytic solution of a vibrating beam with the first mode, a length of $b(x)$, and Dirichlet boundary conditions:

$$\eta(x,y)=\alpha_1\cos(\beta y/b(x))+\alpha_2\cosh(\beta y/b(x)) \tag{A2}$$

for $-b(x)/2\le y\le b(x)/2$, and $\eta(x, y) = 0$ otherwise. Note that α_1, α_2, and β are constant values of 0.8827, 0.1173, and 4.730, respectively. Because the wave number $k(x)$ slowly varies along x as $b(x)$, the waves can be described by the Wentzel−Kramers−Brillouin asymptotic approximation. Under these assumptions, the displacement $w(x, y, t)$ of the BM can be written as

$$w=W(x)\eta(x,y)\exp\left[i\left(\int_0^x k(\xi)d\xi-\omega t\right)\right], \tag{A3}$$

where i and $W(x)$ are an imaginary number and the envelope function, respectively. The distribution of the LRF was determined by analyzing the forward wave as discussed below. In addition, under the fixed boundary condition at the wall of the channel, the solution of the Laplace equation $\nabla^2\varphi_l = 0$ can be described by

$$\varphi_l=\sum_{j=0}^{\infty}A_j\cosh\left[\zeta_j(z+L_2)\right]\cos\left(j\pi y/L_1\right)\exp\left[i\left(\int_0^x k(\xi)d\xi-\omega t\right)\right], \tag{A4}$$

where A_j is the Fourier coefficient for the jth mode. A_j is written as

$$A_j=-2i\omega W(x)\int_{-b(x)/2}^{b(x)/2}\eta(x,y)\cos(j\pi y/L_1)dy\Big/\left[L_1\zeta_j\sinh(\zeta_jL_2)\right] \tag{A5}$$

where $\zeta_j = [k^2(x) + (j\pi/L_1)^2]^{1/2}$. From the average variation principle, the envelope function is described by $W(x)=c\big/\left[\partial f_\omega/\partial k\right]^{1/2}$, where c is a constant. Here, f_ω is represented by the following eikonal equation:

$$f_\omega(k(x))=D\left[C_1k^4(x)-2C_2k^2(x)+C_1\left(\beta/b(x)\right)^4\right]-\omega^2\left[\rho_mhC_1+\rho_l\sum_{j=0}^{\infty}B_j\right], \tag{A6}$$

where $C_1=\int_{-b(x)/2}^{b(x)/2}\eta(x,y)^2dy$, $C_2=\int_{-b(x)/2}^{b(x)/2}\eta(x,y)\partial^2\eta(x,y)/\partial y^2dy$, and $B_j=2\left[\int_{-b(x)/2}^{b(x)/2}\eta(x,y)\cos(j\pi y/L_1)dy\right]^2\Big/\left[L_1\zeta_j\tanh(\zeta_jL_2)\right]$. When an angular frequency ω or frequency $f(=\omega/(2\pi))$ is given, the wave number k is a variable solved using the equation $f_\omega(k) = 0$ (Eq. (A6)) at the

fixed position x and the width b. In fact, the value of position x does not matter for these equations, whereas the width b must be specified. The Newton method was used to solve the equation. The iteration procedure was repeated until the residual became less than a specified tolerance of 10^{-6}. The term, including summation, was derived from j = 0 to 100, which is adequate for this tolerance value. The calculation was performed for the condition with air in the channel, which is referred to as the air condition. The frequency was changed in the range of 1.0–20.0 kHz. Parameters for the calculation were as follows: $D = 1.7 \times 10^5$ Pa·m^4, ρ_m = 1780 kg/m^3, ρ_{air} = 1.2 kg/m^3, L_1 = 17 mm, and L_2 = 4.0 mm.

Characteristic frequencies for the cochlear implant electrode stimulation sites usually are estimated using the Greenwood frequency-position equation (Greenwood, 1990). The original Greenwood frequency position function for the organ of Corti was derived from human frequency resolution integration estimates (critical bandwidths), assuming that critical bandwidths followed an exponential function of distance along the cochlear partition and corresponded to a constant physical position on the BM. Greenwood proposed the following equation for frequency along the organ of Corti: $F = A(10^{ax} - k)$, where F is frequency and x is a portion of the length obtained from the position x from the apical end of the BM to the region of interest normalized based on the total length of the membrane. For example, coefficients for human cochleae are: A = 165.4, a = 2.1, and k = 1.0, whereas those for cat cochleae are A = 456.0, a = 2.1, and k = 0.8 (Greenwood, 1990). The equation allows estimation of the represented frequency based on the cochlear position expressed as a percentage of the total length of the organ of Corti.

Compressive effect. Compression was modeled using a nonlinear function, which is described as

$$y = g_{f_{\mathrm{CF}}}(d) = G\left(A_{f_{\mathrm{CF}}} d + (1 - A_{f_{\mathrm{CF}}}) \frac{d_h}{d_h + |d|} d \right) \tag{A7}$$

where y is an output signal, d is a voltage signal from the ABM output, and G and d_h are constant. Here, $A_{f_{\mathrm{CF}}}$ is a CF-dependent constant value and is written as

$$A_{f_{\mathrm{CF}}} = 10^{\left(a_0 + a_1 x^1 + a_2 x^2 + a_3 x^3\right)} \tag{A8}$$

and

$$x = 33.3 \log_{10}(f_{\mathrm{CF}}) - 83.3, \tag{A9}$$

where f_{CF} is the CF. The parameter values were defined as follows: $G = 5.0$, $d_h = 1.0$, $a_0 = 6.73 \times 10^{-6}$, $a_1 = 2.67 \times 10^{-4}$, $a_2 = -0.103$, and $a_3 = 8.16 \times 10^{-3}$.

Appendix B

IHC model. In the IHC model, the output u of the asymmetric nonlinear function was described as

$$u(d) = \frac{16.5}{1 + \exp\left(-(d - 20)/10.5\right)\left[1 + \exp\left((d - 17.0)/11.6\right)\right]} - 1.78, \tag{B1}$$

where d was the electric output of the ABM. A set of filtering equations was described as

$$dx_1 / dt = -x_1 / \tau_1 + u / \tau_1, \tag{B2}$$

$$dx_2 / dt = x_1 / \tau_2 - x_2 / \tau_2, \tag{B3}$$

$$V_{\mathrm{ihc}} = G(f_{\mathrm{CF}})\left(x_1 - g_0 x_2 + b_0\right), \tag{B4}$$

where V_{ihc} represents the membrane voltage output, $G(f_{\mathrm{CF}}) = 1/\left(\mathrm{pol}\left(\log_{10}(f_{\mathrm{CF}})\right) + g_2\right)$, $\mathrm{pol}(x) = g_1 x^3 + g_2 x^2 + g_3 x + g_4$, and f_{CF} is a CF of the IHC. The parameters were defined as follows: $\tau_1 =$

0.16 ms, $\tau_2 = 0.48$ ms, $b_0 = 0.1$, $g_0 = 0.65$, $g_1 = -0.047$, $g_2 = 0.56$, $g_3 = -2.3$, and $g_2 = 3.1$.

Appendix C

IHC-AN synapse model. The immediate permeability (P_I) was a soft rectifying function of the model IHC response (V_{ihc}) and described using the following equation:

$$P_I(t) = p_1 \ln\,[1 + \exp(34.657 \cdot V_{\text{ihc}}(x,\ t)], \tag{C1}$$

The relationship was linear above the resting permeability and exponentially decayed to 0 for negative inputs. The parameter p_1 determines the immediate permeability at rest and the spontaneous firing rate of the model fiber. In this study, the parameter value of p_1 is 0.0173. For synapses between IHCs and AN fibers, a simple three-store diffusion model was used to introduce neural adaptation (Westerman and Smith, 1988). The three stores C_G, C_L, and C_I are respectively referred to as global, local, and immediate concentrations. The dynamics of C_I and C_L were governed by the following time-dependent differential equations:

$$d\,C_{\text{I}}/dt = \left[-P_{\text{I}}(t)C_{\text{I}} + P_{\text{L}}\left(C_{\text{L}} - C_{\text{I}}\right)\right]/V_{\text{I}} \tag{C2a}$$

$$d\,C_{\text{L}}/dt = \left[-P_{\text{L}}\left(C_{\text{L}} - C_{\text{I}}\right) + P_{\text{G}}\left(C_G - C_{\text{L}}\right)\right]/V_{\text{L}} \tag{C2b}$$

In these equations, the continuous-time version of the original adaptation model used by Carney (1993) was simplified using fixed values for the immediate and local volumes (V_I and V_L in the unit "volume"), the local and global permeability (P_{L} in $s^{-1}$and P_{G} in s^{-1}) (Westerman and Smith, 1988; Lin and Goldstein, 1995), and the global concentration (C_{G} in spikes/volume). The output of the AN synapse model

represents the instantaneous synaptic activity rate($s(t)$) of an individual high-spontaneous-rate-threshold AN fiber; the output is given by $s(t) = R_0 P_I(t) C_I(t)$ (Westerman and Smith, 1988), where R_0 is a scaling parameter and a CF-dependent function such that $R_0 = g(x) = 4.06 \times x^3 - 51.3 \times x^2 + 211 \times x - 280$ and $x = \mathrm{og}_{10}(f_{CF})$, where f_{CF} is a CF.

The Westerman model is usually used as a single channel model, which represents a set of IHC-AN synapses with similar properties (e.g., specific positions on the BM). Here, we extend the description of the single channel to a multichannel version. Equation (C2) is a set of time-dependent first-order differential equations that can be transformed into a state model in a state-space model representation. In addition, the synaptic response or instantaneous firing rate ($s(t)$) can be obtained by multiplying the input $P_I(t)$ by $C_I(t)$; this can also be described as an observation model. Thus, the equations can be transformed into a time-varying state-space model representation, which is given as

$$d\mathbf{x} / dt = P(t)\mathbf{x} + p_0, \qquad \text{(C3a)}$$

$$s = K(t)x + \sigma\xi(t), \qquad \text{(C3b)}$$

where $\mathbf{x} = [C_I(t), C_L(t)]^T$ and the superscript T denotes the transpose of the vector. Moreover,

$$P(t) = \begin{bmatrix} -(P_I(t) + P_L) / V_I & P_L / V_I \\ P_L / V_L & -(P_L + P_G) / V_L \end{bmatrix}, \qquad \text{(C4)}$$

$p_0 = [0, P_G C_G / V_L]^T$, $K(t)=[R_0 P_I(t), 0]^T$, σ is an observed noise intensity, and $\xi(t)$ is the standard Gaussian white noise. Equations (C3a) and (C3b) respectively correspond to a state model and an observation model, and they are the same as Eqs. (1a) and (1b). If the matrix $P(t)$ is regular at any time point, the solution of Eq. (C3a) can be directly obtained as

$$\boldsymbol{x}(t) = \exp\left(\int_0^t P(s)ds\right)\left[\boldsymbol{x}(0) - \int_0^t \exp\left(-\int_0^s P(u)du\right) p_0 ds\right]. \tag{C5}$$

As stated above, the instantaneous release probability of a vesicle from the immediate store ($P_I(t)$) in the Westerman model is usually a function of the intracellular IHC voltage ($V_{\text{ihc}}(x, t)$), which in turn is determined by the input sound stimulus. For a high-frequency tone burst, the IHC receptor potential V_{IHC} is dominated by the "dc" component (Russell and Sellick, 1978; Cheatham and Dallos, 1993). In accordance with previous analyses (Westerman and Smith, 1988; Zhang and Carney, 2005), we hereafter assume that $P_I(t)$ is a constant value (denoted as p_{c2}) after the onset, whereas the value of $P_I(t)$ before the onset is denoted as p_{c1}. In the case of the periodic input of a sound stimulus with a lower-frequency tone burst, we will further discuss this assumption later—i.e., $P_I(t)$ is a time-varying function. Thus, the time-dependent coefficient matrix $P(t)$ in Eq. (C3a) is considered to be a constant matrix after the onset of the stimulus (i.e., $P_I(t) = p_{c2}$ for $t \geq 0$). For additional analysis, Eq. (C3a) can be transformed into the Laplace (complex frequency) domain for $t > 0$ as follows:

$$sX(s) - x(0^-) = PX(s) + p_0 / s \, , \tag{C6}$$

where $x(0^-)$ is the reservoir concentration on the vector before the onset. The solution of Eq. (C6) can be obtained as

$$X(s) = \left[sI - P\right]^{-1}\left[x(0^-) + p_0 / s\right] \tag{C7}$$

under the assumption that the matrix ($sI - P$) is regular. Thus, the time domain solution of $u(t)$ ($t \geq 0$) can be described as

$$\begin{aligned} u(t) &= R_0 P_I(t) C_I(t) + \sigma\zeta(t) \\ &= R_0 P_{c2}\left\{C_0 + C_1 e^{\lambda_1 t} + C_2 e^{\lambda_2 t}\right\} + \sigma\zeta(t) \\ &= \Phi_0 + \Phi_1 e^{\lambda_1 t} + \Phi_2 e^{\lambda_2 t} + \sigma\zeta(t) \end{aligned} \tag{C8}$$

In Eq. (C8), the constant parameters are given as

$$\lambda_1, \lambda_2 = \left(p_{11} + p_{22} \pm \sqrt{(p_{11} - p_{22})^2 + 4 p_{12} p_{21}} \right) \Big/ 2 \quad (\lambda_1 \geq \lambda_2) \tag{C9}$$

and

$$C_0 = P_L P_G C_G \big/ \left(V_I V_L \lambda_1 \lambda_2 \right), \tag{C10a}$$

$$C_1 = \left[\lambda_1 \left(C_I(0^-) - C_0 \right) - \left\{ p_{22} C_I(0^-) + p_{12} C_L(0^-) - (\lambda_1 + \lambda_2) C_0 \right\} \right] \big/ \left(\lambda_1 - \lambda_2 \right), \tag{C10b}$$

$$C_2 = \left[-\lambda_2 \left(C_I(0^-) - C_0 \right) + \left\{ p_{22} C_I(0^-) + p_{12} C_L(0^-) - (\lambda_1 + \lambda_2) C_0 \right\} \right] \big/ \left(\lambda_1 - \lambda_2 \right), \tag{C10c}$$

where

$$\begin{bmatrix} p_{11} & p_{12} \\ p_{21} & p_{2} \end{bmatrix} = \begin{bmatrix} -(p_{c2} + P_L)/V_I & P_L / V_I \\ P_L / V_L & -(P_L + P_G)/V_L \end{bmatrix}, \tag{C11}$$

and

$$C_I(0^-) = \left(-p_{12} P_G C_G / V_L \right) \big/ \left(p_{12} p_{21} + p_{22} (p_{c1} + P_L) / V_I \right), \tag{C12a}$$

$$C_L(0^-) = \left(-(p_{c1} + P_L) P_G C_G / (V_I V_L) \right) \big/ \left(p_{12} p_{21} + p_{22} (p_{c1} + P_L) / V_I \right). \tag{C12b}$$

Equation (C8) indicates that the response is characterized functionally by two exponential components and five constant parameters. In this study, we assumed that the relationship between the rapid adaptation time constant (τ_1) in ms and the CF (f_{CF}) in Hz can be described as

$$\tau_1 = -1.72 \log_{10}(f_{\mathrm{CF}}) + 8.10. \tag{C13}$$

The relationship is directly obtained by linearly fitting the result shown in Fig. 11 of Westerman and Smith (1984), which reflects results from Mongolian gerbils. Because the adaptation time constant is slower (range, 30 ms to 60 ms) for several species (Meddis, 1986b; Zhang and Carney, 2005; Westerman

and Smith, 1985), we assumed a similar linear relationship between the time constants τ_2 and CFs in the logarithmic scale and $\tau_2 = -10.0\log_{10}(f_{CF}) + 90.0$ (ms). This assumption requires further investigation, however. To determine parameters of the model described with Eq. (C3), we assumed CF-independent constant values for six parameters based on the values reported in Heinz et al. (2001). Therefore, because rapid and slow adaptation constants are both CF-dependent values, the other parameters (i.e., P_G and V_I) are also CF-dependent. To determine the model from Eq. (C3) of each channel, we need to determine the other three parameters Φ_0, Φ_1, and Φ_2 from PSTHs.

In addition, hair cells are innervated by two different types of AN fibers (type I and II). Most IHCs connect to type I fibers, whereas OHCs form synapses with type II fibers. A number of studies have detailed the recording of type I fibers, and the characteristics of these fibers are relatively understood. In contrast, it appears to be harder to record from type II fibers, and very little is known about their functional roles (Weisz et al., 2009). Furthermore, anatomic evidence suggests that information sent by OHCs through type II fibers will be slower, less robust, and less specific due to convergent connection patterns between IHCs and type I fibers. Here, therefore, we have focused on modeling only type I fibers. The parameter values were defined as follows: $C_G = 6.67 \times 10^3$ spikes/volume, $P_L = 0.06$ s^{-1}, $P_G = 0.03$ s^{-1}, $V_I = 5.0 \times 10^{-4}$ unit volume, $V_L = 5.0 \times 10^{-3}$ unit volume, $R_0 = 5.0 \times 10^2$, and $\sigma = 5.0$ spikes/s.

Appendix D

Discharge generator. The time-dependent Poisson rate $R(t)$ is described as

$$R(t) = s(t)\left[1 - H(t)\right], \tag{D1}$$

where $s(t)$ is the AN synaptic signal and $H(t)$ is the discharge history. $H(t)$ is determined by a sum of two exponentials (Westerman and Smith, 1985; Zhang et al., 2001):

$$H(t) = \begin{cases} c_0 \exp\left(-(t - t_1)/s_0\right) + c_1 \exp\left(-(t - t_1 - R_A)/s_1\right) & \text{for } (t - t_1) \geq R_A \\ 1.0 & \text{for } (t - t_1) < R_A \end{cases}, \tag{D2}$$

where t_1 is the time of the preceding discharge, and c_0, c_1, s_0, s_1, and R_A are constant parameters: 0.5, 0.5, 1.0 (ms), 12.5 (ms), and 0.75 (ms), respectively. Discharges do not occur during the absolute refractory time R_A, and $H(t)$ varies continuously from 1 to 0 as the interval from the previous discharge increases beyond R_A.
